# Dose-intensive chemotherapy with growth factor or autologous bone marrow or stem-cell transplant support in first-line treatment of advanced or metastatic adult soft tissue sarcoma: a clinical practice guideline

**DOI:** 10.3747/co.v15i2.162

**Published:** 2008-04

**Authors:** S. Verma, J. Younus, A.E. Haynes, D. Stys–Norman, M. Blackstein, the members of the Sarcoma Disease Site Group of Cancer Care Ontario’s Program in Evidence-based Care

**Affiliations:** * The Ottawa Hospital, Ottawa, ON; † London Regional Cancer Centre, London, ON; ‡ Cancer Care Ontario Program in Evidence-based Care, McMaster University, Hamilton, ON; § Mount Sinai Hospital, Toronto, ON; || Please see the Web site of Cancer Care Ontario’s Program in Evidence-based Care (www.cancercare.on.ca/index_AboutthePEBC.htm#dsgg) for a complete list of current Sarcoma Disease Site Group members

**Keywords:** Soft tissue sarcoma, dose-intensive chemotherapy, growth factor, autologous bone marrow or stem-cell transplantation, clinical practice guideline

## Abstract

**Questions:**

- In patients with inoperable locally advanced or metastatic soft tissue sarcoma, does first-line dose-intensive chemotherapy supported by growth factor or autologous bone marrow or stem-cell transplantation improve response rate, time to disease progression, or survival as compared with standard-dose chemotherapy?
- What are the effects of first-line dose-intensive chemotherapy supported by growth factor or autologous bone marrow or stem-cell transplantation on toxicity and quality of life?

**Perspectives:**

Because therapeutic options for adult patients with advanced or metastatic soft tissue sarcoma are scarce and the possibility of cure for these patients is extremely limited, the Sarcoma Disease Site Group (dsg) felt that a review of the available literature on dose-intensive chemotherapy for adult patients with locally advanced or metastatic soft tissue sarcoma and subsequent development of a clinical practice guideline based on the evidence were important.

**Methodology:**

A systematic review was developed and clinical recommendations relevant to patients in Ontario were drafted. The practice guideline report was reviewed and approved by the Sarcoma dsg, which comprises medical oncologists, radiation oncologists, surgeons, a pathologist, a methodologist, and community representatives. External review by Ontario practitioners was obtained through a mailed survey, the results of which were incorporated into the practice guideline. Final review and approval of the practice guideline was obtained from the Report Approval Panel.

**Practice Guideline:**

Based on the systematic review, consensus, and external review, the Sarcoma dsg makes these recommendations:

- Dose-intensive chemotherapy with growth factor support is not recommended in the first-line treatment of patients with inoperable locally advanced or metastatic soft tissue sarcoma.
- The data are insufficient to support the use of high-dose chemotherapy with autologous bone marrow or stem-cell transplantation as first-line treatment in this group of patients.
- Eligible patients should be encouraged to enter clinical trials assessing novel approaches or compounds.

**Qualifying Statements:**

High-dose chemotherapy with growth factor or autologous bone marrow or stem-cell transplantation has adverse effects similar to those seen with standard-dose chemotherapy. With high-dose regimens, the incidence of grades 3 and 4 thrombocytopenia is significantly higher, and neutropenic fever and febrile neutropenia occur more frequently. Compared with standard treatment, the rate of treatment-related death is also higher with high-dose regimens.

## 1. QUESTION

- In patients with inoperable locally advanced or metastatic soft tissue sarcoma, does first-line dose-intensive chemotherapy supported by growth factor or autologous bone marrow or stem-cell transplantation improve response rate, time to disease progression, or survival as compared with standard-dose chemotherapy?
- What are the effects of first-line dose-intensive chemotherapy supported by growth factor or autologous bone marrow or stem-cell transplantation on toxicity and quality of life?

For the purposes of this practice guideline, “dose-intensive chemotherapy” is defined as regimens administered with intent to increase standard doses of chemotherapy with the support provided by the use of either, or both of, hematopoietic growth factors or autologous bone marrow or stem-cell transplantation. “Standard chemotherapy” includes regimens that were previously evaluated in a large phase ii trial or a randomized phase iii trial without growth factor support.

## 2. CHOICE OF TOPIC AND RATIONALE

Treatment of advanced (unresectable or metastatic) soft tissue sarcoma remains a challenging and problematic area in oncology. Most people who present with metastatic disease are not candidates for surgical resection, and consequently, systemic therapy is the only remaining option 1,2. Cytotoxic agents such as doxorubicin and ifosfamide are commonly used and have demonstrated activity in patients with metastatic soft tissue sarcoma. Studies showed that approximately 20%–30% of patients respond to those drugs used as single agents 3–6. In addition, a number of prospective trials demonstrated that growth factors such as granulocyte colony–stimulating factor (g-csf) and granulocyte–macrophage colony– stimulating factor (gm-csf), or autologous bone marrow or stem-cell transplantation may improve hematologic tolerance for dose-intensive combination chemotherapy regimens 7–9.

Because therapeutic options for adult patients with advanced or metastatic soft tissue sarcoma are extremely limited and the possibility of cure for these patients is nearly nonexistent, the Sarcoma Disease Site Group (dsg) felt that a review of the available literature on dose-intensive chemotherapy for adult patients with locally advanced or metastatic soft tissue sarcoma and subsequent development of a clinical practice guideline based on the evidence were important.

## 3. METHODOLOGY

### 3.1 Guideline Development

The present practice guideline was developed by the Sarcoma dsg of Cancer Care Ontario’s Program in Evidence-Based Care (pebc) using the methods of the practice guidelines development cycle 10. This practice guideline is a convenient source of the best available evidence on dose-intensive chemotherapy for patients with inoperable locally advanced or metastatic soft tissue sarcoma. It was developed through systematic review, evidence synthesis, and input from practitioners in Ontario. The systematic review (currently under consideration for publication elsewhere) forms the basis of this report and was used by the Sarcoma dsg to formulate draft recommendations meant to promote evidence-based practice in Ontario. The pebc is editorially independent of Cancer Care Ontario and the Ontario Ministry of Health and Long-Term Care.

External review was obtained for the practice guideline report through a mailed survey of Ontario practitioners. The survey consisted of items addressing the quality of the draft practice guideline report and the recommendations, and asking whether the recommendations should serve as a practice guideline. Final approval of the original practice guideline report was obtained from the pebc Report Approval Panel. All members of the Sarcoma dsg disclosed information on potential conflicts of interest. No conflicts were declared.

### 3.2 Literature Search Strategy

A systematic search of the medline, embase, and Cochrane Library databases for 1980–2006 was conducted for practice guidelines, systematic reviews or meta-analyses, and randomized controlled trials, controlled clinical trials, and phase i, ii, and iii clinical trials. In addition, the 1998–2005 conference proceedings of the American Society of Clinical Oncology (asco) were searched for abstracts of relevant trials. The Canadian Medical Association Infobase (mdm.ca/cpgsnew/cpgs/index.asp) and the National Guidelines Clearinghouse (www.guideline.gov/) were also searched for existing evidence-based practice guidelines.

### 3.3 Results

Two phase iii randomized trials 11,12, twelve phase ii trials 7,13–23, and five phase i dose-escalation trials 24–28 met the eligibility criteria and were included in the systematic review.

In the first randomized trial, patients received either a standard dose of doxorubicin and ifosfamide or a high dose of doxorubicin combined with a standard dose of ifosfamide 11. That trial did not detect any statistically significant differences in overall survival or response rate. The second trial randomized patients to either standard-dose maid [mesna, Adriamycin (doxorubicin: Pharmacia, Kalamazoo, MI, U.S.A.), ifosfamide, dacarbazine] or maid + 25% with g-csf support 12. Survival data have not yet been reported for that trial, and preliminary results suggest no benefit in response rate. Both randomized trials detected higher rates of adverse effects in patients receiving the high-dose regimens.

One randomized phase ii trial 16 that examined the role of high-dose ifosfamide found no statistically significant improvements in overall or disease-free survival between the high- and standard-dose chemotherapy arms. The remaining phase ii trials reported promising response rates and overall median survivals; however, those results were not replicated in the randomized trials.

## 4. DSG CONSENSUS PROCESS

The report was circulated for review and discussion by the Sarcoma dsg, which comprises medical oncologists, radiation oncologists, surgeons, a pathologist, a methodologist, and community representatives. The members conceded that, given the available data, high-dose chemotherapy with growth factor or autologous bone marrow or stem-cell transplantation is not recommended for the routine treatment of patients with inoperable locally advanced or metastatic soft tissue sarcoma.

## 5. INTERNAL REVIEW

The completed report was reviewed and approved by the pebc Report Approval Panel, which consists of two members, including an oncologist with expertise in clinical and methodology issues. The Panel offered these comments:

- A recommendation concerning the use of stem cell transplantation cannot be made given that insufficient data exist.
- The recommendation did not specify the first-line treatment setting.
- Should the phase i and phase ii trials assessing growth factors be included, given the availability of the randomized trials?

To address the key comments, the Sarcoma dsg

- created two separate recommendations, with one stating that a recommendation for the use of bone marrow or stem-cell transplantation could not be made because of insufficient data;
- incorporated “first-line” into the recommendations; and
- noted that inclusion of phase i and phase ii trials was in part a reflection of past practice and in part the result of a desire on the part of the dsg to provide a detailed description of the efficacy and toxicity of the treatments.

Once those key issues were addressed, the document was approved without further changes.

## 6. EXTERNAL REVIEW

The Sarcoma dsg circulated the clinical practice guideline and systematic review to practitioners in Ontario for review and feedback.

### 6.1 Methods

Feedback was obtained through a mailed survey of 74 practitioners in Ontario, including medical oncologists, radiation oncologists, and surgeons. The survey consisted of items evaluating the methods, results, and interpretive summary used to inform the draft recommendations and asking whether the draft recommendations should be approved as a practice guideline. Written comments were invited. The survey was mailed on February 22, 2006. Follow-up reminders were sent at 2 weeks (post card) and 4 weeks (complete package mailed again). The Sarcoma dsg reviewed the results of the survey.

### 6.2 Results

Of the 74 practitioners surveyed, 23 responded (31% response rate). Responses include returned completed surveys and telephone, fax, and e-mail responses. Of the practitioners that responded, 8 indicated that the report was relevant to their clinical practice, and they completed the survey. One practitioner indicated that the topic was relevant, but that practitioner did not complete the questionnaire because of a lack of direct interaction with patients. That practitioner’s comments were therefore not included in the results presented here. Table I summarizes key results of the practitioner feedback survey.

### 6.3 Summary of Written Comments

Of the 8 respondents, 1 clinician provided suggestions for future document development and content. Those suggestions were noted at the pebc office. No other feedback was provided.

## 7. PRACTICE GUIDELINE

The present practice guideline reflects the integration of the draft recommendations with the feedback obtained from the external review process. The guideline was approved by the Sarcoma dsg and the Report Approval Panel.

### 7.1 Recommendations

- Dose-intensive chemotherapy with growth factor support is not recommended in the first-line treatment of patients with inoperable locally advanced or metastatic soft tissue sarcoma.
- The data are insufficient to support the use of high-dose chemotherapy with autologous bone marrow or stem-cell transplantation as first-line treatment in this group of patients.
- Eligible patients should be encouraged to enter clinical trials assessing novel approaches or compounds.

### 7.2 Qualifying Statements

High-dose chemotherapy with growth factor or autologous bone marrow or stem-cell transplantation has adverse effects similar to those seen with standard-dose chemotherapy. With high-dose regimens, the incidence of grades 3 and 4 thrombocytopenia is significantly higher, and neutropenic fever and febrile neutropenia occur more frequently. Compared with standard treatment, the rate of treatment-related death is also higher with high-dose regimens.

## 8. PRACTICE GUIDELINE DATE

This clinical practice guideline is based on work completed in April 2006. All approved pebc clinical practice guidelines are reviewed and updated regularly. Please visit the Cancer Care Ontario Web site (www.cancercare.on.ca) for a complete list of current projects.

## Figures and Tables

**TABLE I:** Responses to eight items on the practitioner feedback survey

Item	Strongly agree or agree	[n (%)] Neither agree nor disagree	Strongly disagree or disagree
The rationale for developing a guideline, as stated in the “Introduction” section of the report, is clear.	7 (100)	0	0
There is a need for a guideline on this topic.	6 (85.3)	1 (14.3)	0
The literature search is relevant and complete.	7 (100)	0	0
The results of the trials described in the report are interpreted according to my understanding of the data.	7 (100)	0	0
The draft recommendations in the report are clear.	7 (100)	0	0
I agree with the draft recommendations as stated.	7 (100)	0	0
This report should be approved as a practice guideline.	6 (85.3)	0	1 (14.3)
	*Very likely or likely*	*Unsure*	*Not at all likely or unlikely*
If this report were to become a practice guideline, how likely would you be to make use of it in your own practice?	6 (85.3)	1 (14.3)	0
